# A Usability Survey of a Quality Improvement Data Visualization Tool among Medical Intensive Care Unit Nurses

**DOI:** 10.1055/s-0044-1782604

**Published:** 2024-04-05

**Authors:** Abigail M. Williams, Claire L. Davis, Margot Bjoring, Kris Blackstone, Andrew J. Barros, Kyle B. Enfield

**Affiliations:** 1 University of Virginia School of Medicine, Charlottesville, Virginia, United States; 2 Division of Pulmonary and Critical Care Medicine, Department of Medicine, University of Virginia Health System, Charlottesville, Virginia, United States; 3 Department of Quality and Performance Improvement, University of Virginia Health System, Charlottesville, Virginia, United States; 4 Department of Nursing, University of Virginia Health System, Charlottesville, Virginia, United States

**Keywords:** data visualization, intensive and critical care, interfaces and usability, dashboard, quality

## Abstract

**Background** Cognitive overload is prevalent among intensive care unit (ICU) clinicians. Data visualization may decrease cognitive load by assisting with data interpretation and task prioritization. We developed the Bundle Board to display real-time data from the electronic medical record (EMR), highlighting opportunities for action in standardized ICU patient care. This study evaluates the practical usability of this data visualization tool among nurses in the ICU.

**Methods** The tool is offered as an application separate from the EMR and was available in the medical ICU for 8 months before we surveyed unit nursing staff. To evaluate usability of the tool, we adapted the Health-Information Technology Usability Scale and included an option to provide open-ended feedback. Survey data were analyzed using quantitative and qualitative methods.

**Results** ICU nurses were invited to participate through email and verbal announcements. Of the potential participants, 38% ( *N* = 47) responded. The survey demonstrated that the tool was perceived as usable. For each subscale, mean scores were as follows: Perceived Ease of Use 4.40, Impact 4.14, User Control 4.07, and Perceived Usefulness 3.61. There were no significant differences between core and contracted nurses or after stratifying by duration of Bundle Board use. Fifteen respondents completed the optional free-text portion of the survey. Qualitative analysis revealed six subthemes focusing on perceived impacts on quality and safety, cognitive burden and workload, and emotional impact of the Bundle Board.

**Conclusion** The Bundle Board demonstrated good usability among ICU nurses, who provided substantive feedback for its improvement. These observations may be generalizable to other comparable interventions. Iterative feedback from end users is vital to developing and implementing a digital health intervention. Our study provides a framework for performing a usability analysis within a specific clinician population and environment.

## Introduction

Cognitive overload is prevalent in the critical care setting due to high patient acuity, frequent interruptions, and large information volume, which may contribute to medical errors. 1 2 Worsening the cognitive load of clinicians, cluttered electronic medical record (EMR) displays are known to worsen clinical performance, an effect made prominent with more complex tasks. 3 Furthermore, poor EMR usability has demonstrated associations with higher rates of burnout and intention to leave among nurses 4 and can hinder communication between members of the interdisciplinary team including intensivists, nurses, and respiratory therapists. 5 6

Bundled care refers to the implementation multiple evidence-based practices that together improve outcomes. 7 Several intensive care unit (ICU) bundles including the ABCDEF Bundle 8 have significantly improved morbidity and mortality. Reminders in the form of checklists and dashboards are commonly employed in ICUs to organize relevant information for standardized or bundled care. 7 9 The dynamic nature of real-time electronic data visualization tools saves time, 10 11 improves clinical decision-making, 11 12 promotes safety protocol adherence, 11 13 14 15 16 and facilitates clinician communication. 11 14 17 Still, data visualization tools are only effective when they address the needs of and integrate within the workflow of target clinicians. 10 12 18 19

The coronavirus disease 2019 pandemic highlighted a need to reinvigorate previous adherence to the ICU bundled care elements. 8 20 We developed a digital display called the Bundle Board to support the documentation, completion, and communication of several bundled care topics, starting with managing invasive catheters. Our group recently published our experience with creating, implementing, and studying the early outcomes of the Bundle Board. 14 We observed increased completion and documentation of device maintenance care and reduced the duration of problematic conditions for invasive catheters in medical intensive care unit (MICU) patients. However, we did not investigate the usability of the Bundle Board.

Usability analysis evaluates an end user's ability to efficiently use an information technology tool for a designated purpose. 21 The current study employs quantitative and qualitative methods to analyze the usability of the Bundle Board by ICU nurses and identify areas for improvement in the tool. We hypothesized that the Bundle Board is a highly usable tool for enhancing the ability of MICU nurses to provide and document complete, timely nursing care.

## Methods

### Setting

Our institution is a tertiary care academic medical center in the Southeastern United States with 631 beds, over 8,000 full-time equivalents, a Level 1 trauma center, a nationally recognized cancer center, and a children's hospital. Our institution uses EpicCare (Epic, Verona, Wisconsin, United States, www.epic.com ) as the EMR. This project was applied to our 28-bed MICU. A total of 123 nurses worked in the MICU during this period, including bedside nurses and clinical nursing leadership. The MICU service had a median census of 33 patients (interquartile range = 6) during the implementation phase of the Bundle Board. Some patients are roomed outside of the physical MICU due to capacity constraints. The census was stable throughout the survey period.

### Intervention

The Bundle Board is a data visualization tool for standardized interventions that reduce ICU morbidity and mortality. The development, implementation, and early clinical outcomes of the Bundle Board are described in a separate manuscript. 14 The intervention was developed with the input of an interprofessional team including nurses, physicians, and clinical informaticists. 14 The Bundle Board is displayed on four wall-mounted screens distributed throughout the secured MICU. It is also available as an application on computer workstations.

**Fig. 1:**
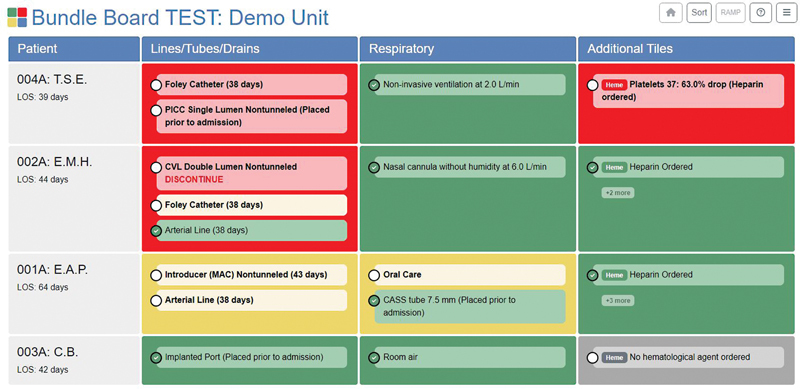
Unit overview. This is a test environment with no patient information. Three quality-based patient care topic columns are included on the unit overview: lines/tubes/drains (invasive catheters), respiratory care, and additional tiles (which address hematologic care.) Each tile is shown as red, yellow, green, blue, or gray. Red tiles signify documented findings that need attention. Yellow tiles reflect either incomplete or undocumented care. Green tiles mean all appropriate care has been provided and documented, and there are no concerning findings. Lastly, blue tiles convey that the patient is receiving end-of-life care, which has different nursing care priorities. Of note, the gray tiles pictured in this figure were added after the survey, due to nursing feedback. Patient rows with red and yellow tiles automatically float to the top of the unit-based display, promoting collective team awareness of concerning features.

**Fig. 2:**
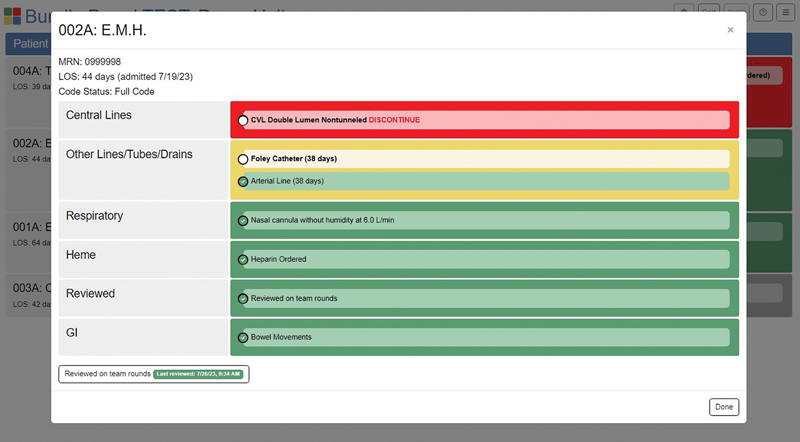
This is a test environment and does not reflect real clinical data. An individual patient's information can be viewed by clicking on the tile containing their initials and room number. The end user can click anywhere outside of the pop-up window, or on the close window button in the upper left corner, to return to the unit overview screen.

**Fig. 3:**
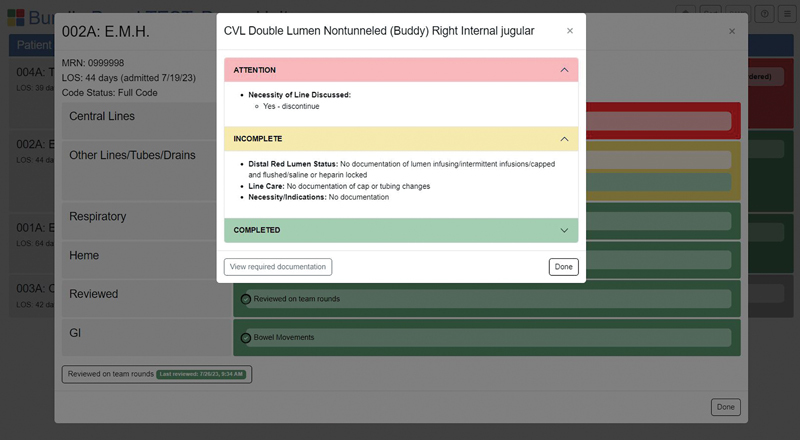
Tile details and color changes, viewed for a sample patient in the test environment. Details surrounding the care item can be viewed by clicking a tile. This will display reasons for the tile's color, as well as any incomplete documentation. Completed work can be viewed with an additional click. The end user can click anywhere outside of the pop-up window, or on the close window button in the upper left corner, to return to the unit overview screen. In this example, a hemodialysis catheter is appearing as a red tile, highlighting that the patient's chlorhexidine gluconate (CHG) bath is overdue. Additionally, the patient is lacking documentation for the necessity and indication. After the CHG bath is administered and documented, the tile would turn from red to yellow, as it is still missing necessity and indication. If the necessity and indication were simultaneously documented at the same time as the CHG bath, the tile would turn from red to green.

The Bundle Board has three quality-based patient care topic columns: maintenance care for invasive catheters, maintenance respiratory care, and laboratories and orders reflecting hematologic care ( Fig. 1 ). Each tile represents a care topic as documented in the EMR and is shown as red, yellow, green, or blue to match standard operating procedures ( Figs. 2 and 3 ). Red tiles highlight documentation that indicates the patient has a condition that “needs attention.” Yellow tiles represent care has either not been performed or not been documented. Green tiles represent care and documentation are complete and without problematic conditions. Blue tiles represent when a patient's care goals are focused exclusively on end-of-life, comfort-focused care. Patient rows with red and yellow tiles automatically float to the top of the unit-based display, promoting collective team awareness of concerning features. The board also displays each patient's length of stay and Sequential Organ Failure Assessment Score, a mortality prediction score, with their name and room number. 22

On the back end, the Bundle Board is built as a module running on an in-house platform that generates early warning scores and time-sensitive information for hospitalized patients using Epic Interconnect Application Programming Interfaces data. The platform provides near real-time access to patient vital signs, laboratories, medications, and flowsheet histories directly from the EMR. The Bundle Board uses specific elements of those data to assess the completion of required documentation and standardized, bundled care goals within specified intervals. The Bundle Board's clinician-facing display refreshes its display every 3 minutes. Patient Protected Health Information is protected behind the University of Virginia (UVA) Health firewall and inaccessible to anyone outside the UVA Health network. Access to the Bundle Board is managed through directory security groups, and users must log into the application separately from the EMR. End users include physicians, nurses, and pharmacists.

### Survey Instrument Development

We adapted the Health Information Technology Usability Scale (Health-ITUES) to assess usability. The Health-ITUES is a validated instrument for quantitative usability assessment of tools specific to the health care setting. 23 24 It has been utilized in other studies examining the usability of patient care dashboard displays. 18 25 26 The Health-ITUES assesses four domains: (1) Quality of Work Life/Impact, (2) Perceived Usefulness, (3) Perceived Ease of Use, and (4) User Control. 23 Responses are quantified using five-point Likert items with a minimum score of 1 corresponding to “strongly disagree” and a maximum score of five relating to “strongly agree” with higher scores indicating higher usability. Items were adapted to reflect our target users (MICU nurses) and the task of delivering ICU care with the Bundle Board. The adapted survey items are available in Table 2 . Participants were invited to offer free-text responses at the end of the survey. All questions were optional.

While we can determine who has logged into the application on individual workstations, we cannot capture who has used the large display screens, which do not require a log-in. Therefore, we were dependent on participants to self-report the duration for which they had used the Bundle Board. We also asked participants to self-identify as contracted (nurses employed by an independent staffing agency) or core staff (nurses directly hired by our institution).

### Recruitment and Enrollment

All nurses working in the MICU were eligible to participate. Potential participants were approached 8 months after the Bundle Board was deployed in the MICU. Participants were recruited via email. Our research team sent a weekly reminder via email and made verbal announcements at nursing sign-outs. Surveys were administered electronically and were open for a predetermined 2-month period. Survey participation was anonymous and voluntary; no compensation was provided. Our Institutional Review Board approved the study (HSR-SBS 5578.)

### Analysis

Quantitative survey item responses were analyzed for mean, median, and standard deviation (SD). Given our customization, Cronbach's α was calculated to measure internal consistency for each subscale. We used the Kruskal–Wallis test to analyze potential covariates, with statistical significance set at *p* < 0.05. We imputed missing data from participants based on their subscale averages to allow for these analyses. Imputed data were excluded from the reported subscale scores. Statistical operations were performed using R Studio. 27

After the survey closed, survey responses were downloaded and imported into Dedoose qualitative data analysis software. 28 Two independent coders assigned codes to every free-text response to perform thematic analysis. 29 Individual codes were grouped into broader categories, and thematic saturation was achieved with no additional topics identified by either coder. The codebook was refined until the intercoder agreement reached a pooled Cohen's kappa statistic of 0.85. Any discrepancies were reviewed and discussed to bring all coding into agreement.

## Results

### Survey Participation

Thirty-eight percent of potential participants responded to the survey ( *N* = 47). Of the respondents, 85% were core staff ( *N* = 40). Nine percent of participants had <1 month of Bundle Board use, followed by 4% with 1 to 3 months, 26% with 3 to 5 months, 17% with 5 to 8 months, and 45% with 8 to 12 months. Four participants omitted between one and nine questions of the 21-question survey. Table 1 shows the distribution of these respondents by length of Bundle Board use and staffing status.

**Table 1:** Respondent demographics

		Length of bundle board use					
		<1 mo	1–3 mo	3–5 mo	5–8 mo	8–12 mo	
Staffing status	Core (Directly hired by institution)	0	2	11	7	20	40 (85%)
	Contracted (Hired through staffing agency)	4	0	1	1	1	7 (15%)
		4 (9%)	2 (4%)	12 (26%)	8 (17%)	21 (45%)	47 (100%)

Abbreviation: Health-ITUES, Health Information Technology Usability Scale.

### Health-Information Technology Usability Scale Survey Results

**Fig. 4:**
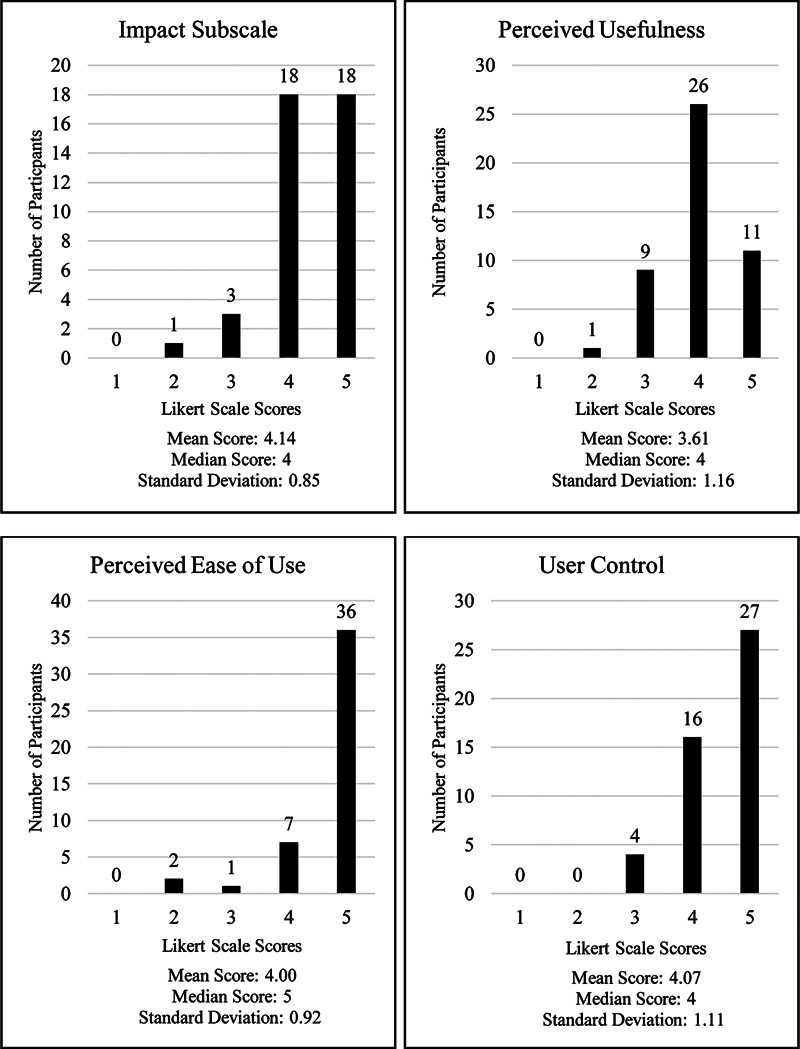
Distribution of Health-ITUES Subscale Scores. Health-ITUES, Health Information Technology Usability Scale.

The mean Health-ITUES score was 3.94 (SD = 1.11). Fig. 4 displays subscale score results and averages, medians, and standard distribution. Health-ITUES subscale scores are shown in Table 2 . All subscales demonstrated appropriate internal agreement (Cronbach's α = 0.88, 0.86, 0.90, 0.87, respectively). There were no statistically significant differences in response distribution between groups of users who had utilized the Bundle Board for different lengths of time ( *p* = 0.37) or between contracted and core nursing staff responses ( *p* = 0.50).

**Table 2:** Health-ITUES Subscale Results

	Survey item	Item mean, median (SD)
Impact	I think the Bundle Board is a positive addition for ICU patients	4.17, 4 (0.87)
	The Bundle Board is an important part of providing critical care	4.00, 4 (0.89)
	I think the Bundle Board improves the quality of care for ICU patients	4.26, 4 (0.77)
Perceived usefulness	Using the Bundle Board makes it easier to manage patients in the ICU	3.64, 4 (1.14)
	Using the Bundle Board enables me to provide care for ICU patients more quickly	3.02, 3 (1.24)
	Using the Bundle Board makes it more likely that complete central line and Foley care is provided for an ICU patient	4.13, 4 (0.93)
	Using the Bundle Board makes it more likely that the complete ICU checklist is performed	3.98, 4 (1.13)
	Using the Bundle Board is useful for critical care patient management	4.09, 4 (0.94)
	I think the Bundle Board presents a more equitable care process for ICU patients	3.87, 4 (0.88)
	I am satisfied with the Bundle Board as a tool in the ICU	3.78, 4 (1.11)
	I provide more complete patient care in a timely manner because of the Bundle Board	2.96, 3 (1.28)
	Using the Bundle Board increases my ability to provide ICU patient care	3.20, 3 (1.17)
	I am better able to provide patient care whenever I use the Bundle Board	3.43, 4 (1.09)
Perceived ease of use	I am comfortable with my ability to use the Bundle Board	4.50, 5 (0.91)
	Learning to use the Bundle Board is easy for me	4.43, 5 (0.86)
	It is easy for me to become skillful at using the Bundle Board	4.24, 5 (0.97)
	I find the Bundle Board easy to use	4.24, 4 (1.02)
	I can always remember how to find and use the Bundle Board	4.61, 5 (0.83)
User control	The Bundle Board gives messages that clearly tells me what additional patient care is needed	4.15, 5 (1.15)
	Whenever I make a mistake using the Bundle Board, I can correct my screen easily and quickly	3.91, 4 (1.13)
	The information (such as care items that need attention and what other documentation is needed) from the Bundle Board is clear	4.14, 4 (1.06)
Average, median, standard deviation of survey responses		3.94, 4 (1.11)

Abbreviations: ICU, intensive care unit; Health-ITUES, Health Information Technology Usability Scale; SD, standard deviation.

Notes: The table above has all statements used for the Health-ITUES survey. All questions were optional. Participants were asked to respond to these statements with the following options: “Strongly agree,” “Somewhat agree,” “Neither agree or disagree,” “Somewhat disagree,” “Strongly disagree.”

The subscale “Perceived Usefulness” had the lowest average domain, which was driven in part by two items, both evaluating time to provide care: “I provide more complete patient care in a timely manner because of the Bundle Board” (mean = 2.96, SD = 1.28) and “Using the Bundle Board enables me to provide care for ICU patients more quickly” (mean = 3.02, SD = 1.24). However, users also reported that “Using the Bundle Board makes it more likely that complete central line and Foley care is provided for an ICU patient” (mean = 4.13, SD = 0.93) and “I think that the Bundle Board improves the quality of care for ICU patients” (mean = 4.26, SD = 0.77).

The subscale “Perceived Ease of Use” had the highest average score, with the most users reporting they found the Bundle Board to be accessible and easy to learn, and they were comfortable using the tool.

### Free-text Results

Of 48 survey participants, 15 submitted free-text responses. Most responses (13/15) were from core staff and came from nurses with 8 to 12 months of experience. Of the 15 free-text responses, six subthemes encompassing perceived impact on quality and safety, changes to cognitive burden and workload, and the emotional impact of the Bundle Board were identified. Responses offering suggestions were also identified. Many responses (10/15) expressed comments that spanned several themes. Table 3 presents each category of free-text responses with subtheme descriptions, examples, and frequency.

**Table 3:** Qualitative themes, descriptions, and examples

	Description	Representative excerpts	Number of respondents *N* (%)
Perceived impact on quality and safety			
Improvements to quality and safety	Improves the quality of care and patient safety	“I think the Bundle Board is a useful tool in looking at the big picture of all of the patient's and ensuring the team is on the same page as to what interventions are in place for the patient…”	10 (67%)
Concerns about quality and safety	Misleading regarding corrective actions in care; limitations of the Bundle Board	“The bundle board provides evidence that things are charted, but that is different than what is actually always occurring at the bedside”	5 (33%)
Bundle Board inaccuracies	Displaying inaccurate or outdated information	“One discrepancy I've noticed—occasionally a red box prompting the need for a foley order will still appear even once a foley order is placed”	5 (33%)
Changes to cognitive burden and workload			
Decreases cognitive burden	Decreases cognitive load, improves collective awareness of individual and unit conditions	“…I think the bundle board has developed into a usable information center to quickly get a sense of patients needs”	5 (33%)
Increases workload	Requires time and energy to manage	“I…sometimes feel that we are chasing numbers instead of focusing on each individual patient”	4 (27%)
Feelings generated from use			
Negative framing	Feelings that the Bundle Board is used for punishment, or helplessness to meet specified conditions	“Even though we're told by charge and admin staff that red tiles are not necessarily punitive, it does feel that way”	8 (53%)
Suggestions			
Suggestions	Ideas or recommendations for the Bundle Board's future	“Should be a way to either clear red tiles or add a comment if it has been addressed and is just unable to be fixed”	11 (73%)

Notes: At the end of the survey, participants were asked, “Do you have any additional feedback?” and had space for free-text response. The table above describes themes expressed by survey participants, as identified by independent coders through qualitative analysis.

Ten of the 15 participants who shared free text (10/15) had statements reflecting a perceived improvement in quality and safety. One participant shared (the Bundle Board is) “a useful tool in looking at the big picture of all of the patients and ensuring the team is on the same page as to what interventions are in place for the patient.” In contrast, five participants shared concerns about negative impacts on quality and safety, including that documentation is not always true to what is happening with actual care: “The bundle board provides evidence that things are charted, but that is different than what is actually always occurring at the bedside.” Increases in cognitive burden and workload were described by four respondents in their free text. For example, “I… sometimes feel that we are chasing numbers instead of focusing on each individual patient,” suggests checking the Bundle Board's requirements detracts nursing energy from bedside care. Additionally, there were some concerns about inaccuracies in the information displayed. One nurse noticed a discrepancy where “…occasionally a red box prompting the need for a Foley order will still appear even once a Foley order is placed.” Eleven of 15 respondents had suggestions for how to improve the Bundle Board with future versions.

## Discussion

### Major Findings

Several groups have explored using data visualization to reduce cognitive overload in the ICU. Our tool is different from many other ICU dashboard tools as it covers multiple elements of standardized ICU care (from the ICU A-F Bundle 8 ), as opposed to gathering information for multidisciplinary rounds, monitoring of patient laboratories and vitals, or addressing one specific clinical goal; additionally, it is not embedded in the EMR. 10 13 15 30 31 Pageler et al 15 developed a patient-specific, EMR-integrated checklist and a unit-wide dashboard with colors to improve compliance with central line-associated bloodstream infection (CLABSI) prevention care; however, providers also had mixed perceptions of the clinical impact. We previously reported clinical outcomes associated with the Bundle Board 14 ; we demonstrate here the tool is perceived as user-friendly and clinically impactful, although it has opportunities for improvement. The findings of our usability assessment highlight the complex interplay between new technology and institutional cultural context through the lens of MICU nurses. 32

Wisner et al 33 identified EMR organization increases the challenge of sharing information effectively and increases cognitive load while delivering nursing care. Some of our central aims with the Bundle Board were to mitigate these barriers. Khasnabish et al 18 highlighted widespread access and clear visualization pathways are critical for successful implementation of data visualization tools. We attempted to accomplish this via Bundle Board availability on large display screens and individual workstations and achieved good “Ease of Use” and “User Control.” This access and visual display may be how the Bundle Board increases situational awareness and helps prioritize tasks—by reducing the burden of finding information and the cognitive load required for identifying essential information. This is also supported by the high Health-ITUES scores for items relating to quality improvement, complete delivery of care for invasive catheters, and free-text responses describing increased team communication. By readily displaying and prioritizing information, the Bundle Board provides a practical example of how data visualization can reduce cognitive workload in ICU nurses.

Because the Bundle Board does not impact the assigned workload—it merely displays what is expected for patient care from MICU leadership, we did not anticipate lower scores on items related to the time required to deliver care. We hypothesize that by highlighting the work to be done, the Bundle Board may contribute to increased perceived workload and perceived time needed for care. Free-text statements support this idea, with one user sharing, “The bundle board serves as a good reminder to perform care at the bedside, but it does not ensure care is done.” In this way, the Bundle Board personifies the conflict between the bedside nurse's workload and the hospital leadership's desire for documentation and data in pursuing quality improvement. Our team is working to streamline documentation and reduce nursing documentation burden. Our survey did not ask nurses to distinguish themselves as bedside nurses or nursing leaders. If our hypothesis is true, these two groups of nurses may have different perceptions of the Bundle Board. Determining perceptions between the bedside nurse and nursing leadership will be essential to explore in the future.

We identified a lower Health-ITUES score for “Whenever I make a mistake using the Bundle Board, I can correct my screen easily and quickly.” We theorize this is because mistakes in EMR documentation appear on the Bundle Board, but nurses must again log into the EMR and go through flowsheet documentation and address one or more specific rows to bring documentation into compliance. While we could develop and update the tool more readily because it is outside the EMR, this survey finding may signal end users prefer EMR-integrated applications. Creating a more seamless integration between the Bundle Board and the EMR could improve these scores and help to further reduce effort in identifying and documenting patient care needs.

### Limitations

The Bundle Board has several usability constraints identified during development and deployment but not captured in the survey. One limitation is that the tool is based on color schemas and may be less clear to people with color blindness. During development, we used a color blindness simulator to help identify colors that may be better than others. 34 Still, the tool could be misinterpreted with certain color blindness ( Supplementary Fig. S1 (available in the online version)). 34 An icon not dependent on color could further signal patient care that needs attention to clinicians with color blindness. We also recognize there may be confusion surrounding the color blue, as it is used within the Bundle Board. Blue signs are used at our institution to identify patients receiving comfort-focused care and during the Bundle Board's development, our nurses voiced these patients should also be identified with blue tiles. While these colors may suit our MICU, they may not be generalizable to other ICUs or institutions. Lastly, the tool does not have a formal strategy for escalation, if red tiles are not addressed. Currently, the application tackles nonemergent content, allowing the tool to be noninterruptive. If we expand the tool to address more emergent care topics, linking the tool with alerts to medical providers or nursing leadership to facilitate escalation may become necessary.

Our study methodology has significant limitations. At the time of study, the Bundle Board Bundle had only been implemented in the MICU. Participation was based on voluntary survey responses, so the responding sample may hold stronger or different opinions than the population of MICU nurses. A response rate of 38%, while in line with other surveys of health care workers, 35 may not be representative of all unit nurses. Furthermore, qualitative analysis was based on the responses of a smaller subset of respondents. Nuances of nurse roles, such as whether the respondent held a leadership role, were not distinguished. Duration of use was based on self-report, and intensity or frequency of use was not assessed. While we can determine who has access and has logged into the application, many nurses primarily use the large display screens that do not require a log-in. Therefore, exposure to the tool cannot be objectively measured.

There are also weaknesses with the Health-ITUES survey itself. For interpretation of the Health-ITUES, we are aware of a single publication describing a “cutoff” of 4.3, below which a population of patients used an eHealth application less often; however, this was not validated among a health care provider population or with a data visualization tool. 36 We hope to mitigate the limitations of the Health-ITUES by comparing our results with subsequent Bundle Board versions. Lastly, reports of workflow improvements or detractions due to the Bundle Board were analyzed qualitatively but were not directly measured.

### Unintended Consequences and Lessons Learned

There is room for improvement in the Bundle Board's usability and acceptability among MICU nurses. Participants in the survey shared concerns that documentation and Bundle Board display do not always equate to bedside care, either via lack of documentation or inaccuracies with the Bundle Board. Documentation is a commonly missed nursing care activity, possibly because of complex patient care and turbulent workflow in the ICU. 37 38 This may be a limitation inherent to a tool based on documentation. Regarding data inaccuracies, the Bundle Board's data come directly from charting, laboratory results, and orders within the EMR. Where most data updates within 3 to 5 minutes, at the time of the survey, there were a few data types dependent on different information streams requiring longer to update. To address these concerns, we have since removed data elements that do not update within 3 to 5 minutes. This is not unique to this data visualization tool, cementing the need for continuous technical support and updates. 25

While we used the culturally symbolic color red to draw attention to concerning findings, we did not anticipate nurses may interpret red tiles as punitive. 39 Representative of this sentiment, one nurse shared, “Even though we're told by charge and admin staff that red tiles are not necessarily punitive, it does feel that way.” This has not been previously reported in usability studies on dashboards using a similar color scheme. 25 As we implement the Bundle Board in other ICUs, we are emphasizing that red tiles are not reflective of the quality of the bedside nurse's care, but instead highlight opportunities for improvement with the goal of collective awareness. During the early implementation stage, many nurses voiced being able to acknowledge red tiles and turn them into a distinct color would be helpful. Based on this feedback, we developed a feature allowing users to mark when the interdisciplinary team has reviewed and acknowledged a care item, changing the tile color from red to gray. The gray tile feature went “live” 2 days after closing the survey. We hope to study the gray tile's impact on the end user experience and team dynamics soon.

As mentioned previously, the ability to correct mistakes had a lower usability survey score. This indicates a greater need for end user support with the tool. With the initial deployment, frequent messaging and coaching were available in the MICU to provide immediate end user support. Later in implementation, education was more limited to electronic handouts and a 5- to 10-minute overview during onboarding for new nurses. We are developing a brief video tutorial to reference during onboarding and within the application to help address the need for additional end user support.

### Next Steps

One of our primary objectives has been to create a complete digitized ICU checklist that incorporates the full ABCDEF Bundle and other standardized care interventions for critically ill patients, such as sedation, analgesia, and delirium management. We hope this will improve the perceived usefulness of the intervention. This work is actively in progress. With the increasing complexity of content on the Bundle Board, it is essential to maintain or improve good usability. We will assess progressive Bundle Board versions with repeated usability assessments and will complement this analysis with semi-structured user interviews and heuristic evaluations from a larger sample of end users and other critical care contexts. 19 32 40 The benefits and drawbacks of the Bundle Board will also be significantly complemented by further patient outcomes analysis, such as its impact on hospital-acquired infections and venous thromboembolism rates.

## Conclusion

Previous studies have shown data visualization dashboards improve adherence to quality guidelines. 11 13 14 15 16 Still, health information technology can negatively impact care quality and workload if it has poor usability. 25 41 42 This study provides a practical quantitative and qualitative analysis of nurses' perceived usability for a novel data visualization tool. Our survey showed the tool to be usable to our MICU nursing group and highlighted positive impacts and areas for improvement of the Bundle Board implementation. Particularly, notable takeaways include the negative effect of a visible checklist potentially increasing cognitive load when not fully integrated within the EMR. Overall, the Bundle Board was perceived to be a usable tool among MICU nurses in providing critical care; their feedback will facilitate continued tool development with a focus on the end user.

## Clinical Relevance Statement

This study provides a usability analysis for a novel data visualization tool in the ICU setting; our research could be replicated among other digital health tools and amplify the use of the Health-ITUES survey instrument. We identified opportunities for improvement and successes that may be generalized to other interventions.
