# Supplementary material for: A Usability Survey of a Quality Improvement Data Visualization Tool among Medical Intensive Care Unit Nurses
Source: ACI open. 2024 Apr 5;8(1):e33–42. doi: 10.1055/s-0044-1782604 (PMC12303040; doi:10.1055/s-0044-1782604)
Supplement: Supplementary file 1 — Supplementary Material [file 10-1055-s-0044-1782604-s202305cr0008.pdf]

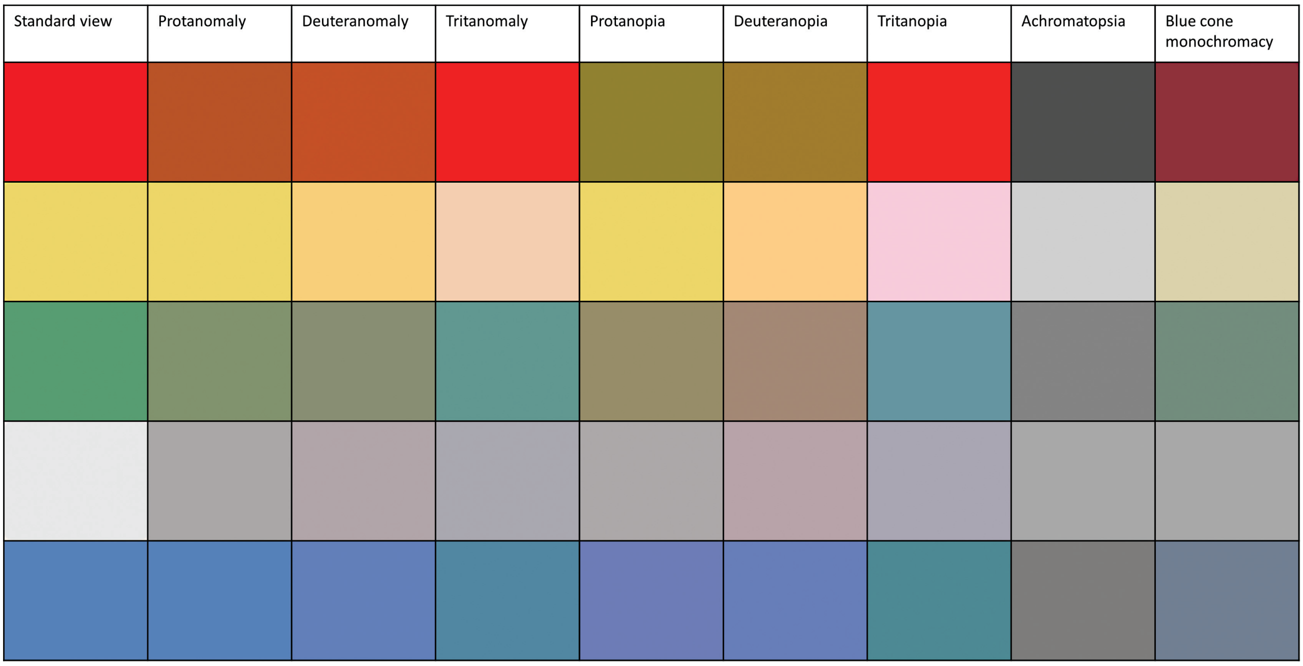

Supplementary Fig. S1 Colors used in the standard view of the Bundle Board, with filters applied to mimic the way people with color blindness might see the Bundle Board.

 © 2024. The Author(s).
